# Geochemical Patterns and Human Health Risks of Less-Regulated Metal(loid)s in Historical Urban and Industrial Topsoils from Alcalá de Henares, Spain

**DOI:** 10.3390/jox16010017

**Published:** 2026-01-21

**Authors:** Antonio Peña-Fernández, Manuel Higueras, Gevorg Tepanosyan, M. Ángeles Peña Fernández, M. C. Lobo

**Affiliations:** 1 Department of Surgery, Medical and Social Sciences, Faculty of Medicine and Health Sciences, University of Alcalá, Ctra. Madrid-Barcelona, Km. 33.600, 28871 Alcalá de Henares, Madrid, Spain; 2 Scientific Computation Research Institute (SCRIUR), Universidad de La Rioja, 26006 Logroño, La Rioja, Spain; 3 The Centre for Ecological-Noosphere Studies, National Academy of Sciences, Abovyan-68, Yerevan 0025, Armenia; 4 Departament of Biomedical Sciences, Faculty of Pharmacy, University of Alcalá, Ctra. Madrid-Barcelona, Km. 33.6, 28871 Alcalá de Henares, Madrid, Spain; 5 Departamento de Investigación Agroambiental, Madrid Institute for Rural, Agricultural and Food Research and Development (IMIDRA), Finca el Encín, Ctra. Madrid-Barcelona, Km. 38.2, 28800 Alcalá de Henares, Madrid, Spain

**Keywords:** urban soils, less-regulated metal(loid)s, platinum group elements, silver enrichment, human health risk assessment, Spain

## Abstract

Nine technology-related metal(loid)s (Ag, Co, Fe, Mo, Pt, Rh, Sb, Se and Y) were monitored in 137 topsoil samples from urban parks, industrial areas and gardens in Alcalá de Henares (Spain) using ICP–MS. Selenium was not detected, while Mo, Sb and Rh showed a high proportion of values below the detection limit, indicating generally low contamination. In contrast, Fe, Co and Y were detected in all samples, with industrial soils showing about two-fold higher median Co and Fe than urban soils. Garden soils displayed marked silver enrichment (median 0.439 vs. 0.068 mg kg^−1^ in urban soils), with Ag pollution indices up to 71 and enrichment factors up to 69; around 17% of garden samples exceeded *EF* > 40, and more than one-quarter had *EF* > 10. Principal component analysis suggested a predominantly geogenic association for Co, Fe and Y and an anthropogenic component for Ag, Mo, Rh and Sb, while Pt was mainly linked to vehicular emissions. Under standard US EPA exposure scenarios applied to the 2001 topsoil concentrations, oral and inhalation hazard quotients for elements with available benchmarks remained <0.2 and inhalation cancer risks for Co were ≤2.5 × 10^−7^, indicating low estimated risk within the model assumptions. However, quantitative risk characterisation remains constrained by benchmark gaps for Pt and Rh and by limited consensus toxicity values for Y, which introduces uncertainty for these technology-related elements. These results should therefore be interpreted primarily as a baseline (2001) in surface soils for Alcalá de Henares rather than as a direct representation of current exposure conditions.

## 1. Introduction

Recent surveys estimate that the health of 60–70% of soils in the European Union (EU) is at risk due to unsustainable management practices and suboptimal conditions. The European Commission’s Joint Research Centre has identified approximately 2.8 million potentially contaminated sites across twelve EU countries and neighbouring states [1]. In response, the EU Action Plan “Towards a Zero Pollution for Air, Water and Soil” was launched in 2021 to establish a framework for regular soil pollution monitoring and to achieve the long-term goal of reducing soil pollution by 2050. In parallel, the European Soil Strategy for 2030 recognises soil contamination as a key priority and has laid the groundwork for the forthcoming Soil Health Law, aimed at ensuring that all EU soils are in a healthy condition by mid-century [2].

Urban soils can serve as a direct or indirect pathway of human exposure to different contaminants, particularly for children and individuals engaged in gardening or recreational activities [3,4]. In Spain, risk assessment studies on urban soils remain limited, and existing research has primarily focused on industrial hotspots or areas near point sources of pollution [5,6,7]. Urban soil investigations are especially scarce in the Comunidad de Madrid, one of the country’s most densely populated regions [8], despite the critical role of urban green spaces in supporting mental wellbeing, public health and social cohesion. Assessing soil contamination in these environments is therefore essential to protect both human and ecosystem health [9].

Alcalá de Henares, a historic and densely populated city within this region, was selected as the focus of this study. Although metal contamination in urban soils remains largely unregulated at the EU level, these soils are increasingly impacted by anthropogenic activity, and available data on their contamination profiles are limited [10]. Moreover, the influence of land use on the spatial distribution of metal(loid)s remains poorly understood [11], despite clear links between urbanisation, land use change and increased contamination risk. Understanding the sources, spatial patterns and potential health implications of metal(loid)s in urban topsoils is vital for informed land management, sustainable urban planning and targeted risk mitigation.

In parallel, quantitative approaches for pollution assessment, source analysis and health risk characterisation for soil metal(loid)s have advanced considerably. Recent studies have moved beyond simple indices to place greater emphasis on site-specific natural background values, multivariate statistics and explicit links with human health risk models. For example, Cai and colleagues developed a partial least squares regression (PLSR) approach to derive dynamic, site-specific natural background values for soil metal(loid)s, improving discrimination between natural and anthropogenic contributions [12]. In subsequent work, they highlighted the importance of combining local geochemical baselines, spatial analysis and health-risk assessment to obtain more policy-relevant evaluations of soil contamination [13]. Jiang et al. [14] further demonstrated that health risk quantification can be refined by considering potentially hazardous elements from the perspective of their emission sources rather than relying solely on bulk soil concentrations. Collectively, these advances underline the value of using robust geochemical baselines, multivariate statistics and explicit health-risk metrics to refine the interpretation of metal(loid) contamination in soils—an approach that we follow here by integrating local background values, pollution indices, principal component analysis and standard human health risk assessment.

Despite this progress, most applications have focused on a relatively narrow set of well-regulated metals (e.g., Pb, Cd, As, Cr, Ni, Cu, Zn) in agricultural or mining-impacted soils, whereas less-regulated, technology-related elements remain comparatively understudied, particularly in urban environments. These include silver (Ag), cobalt (Co), iron (Fe), molybdenum (Mo), antimony (Sb), selenium (Se), yttrium (Y) and platinum group elements (PGEs) such as platinum (Pt) and rhodium (Rh). Many of these elements are critical for advanced technologies, including electronics, catalytic converters, medical devices and renewable energy systems, and increasing production and use are expected to drive greater environmental releases. Although Pt, Rh and Y are often regarded as emerging contaminants due to their expanding use and limited regulatory oversight, most environmental monitoring programmes still do not routinely include them, and toxicological data remain incomplete—particularly for PGEs and rare earth elements.

Spain illustrates these knowledge gaps. National and regional regulations, such as Royal Decree 9/2005 [15] and Order 2770/2006 [16], define generic reference values mainly for classical toxic metals and metalloids in soils, whereas technology-related elements (e.g., Pt, Rh and Y) have not yet been systematically incorporated into regulatory frameworks. Existing urban soil studies in Spain, and specifically in the Comunidad de Madrid, have largely focused on conventional traffic-related metals and metalloids, leaving the occurrence, spatial distribution and potential health risks of less-regulated metal(loid)s in public green spaces and garden soils poorly characterised.

The present study addresses these gaps by investigating nine less-regulated and technology-related metal(loid)s (Ag, Co, Fe, Mo, Pt, Rh, Sb, Se and Y) in topsoils from urban parks, industrial areas and gardens in Alcalá de Henares. Here, we address this gap by providing a land-use-resolved assessment of these under-monitored elements (including Pt and Rh) in urban topsoils, generating a baseline dataset that enables source-informed interpretation and future temporal comparison. In this study, Fe is treated in a dual manner. First, it is used as a predominantly lithogenic element to contextualise soil geochemistry and support normalised interpretations (e.g., Fe-normalised comparisons for Y). Second, because Fe can also be elevated by anthropogenic inputs in industrial/urban settings and is included in Spanish soil screening frameworks, we additionally evaluate Fe concentrations against available guidance values as a pragmatic indicator of potential concern. Garden soils were explicitly included because they are frequently accessed by residents, may be used for food production and recreational activities and have rarely been evaluated for these elements. We therefore combine land use stratification with geochemical mapping, enrichment factors, pollution indices, multivariate analysis (with appropriate treatment of censored data) and human health risk assessment to clarify spatial patterns and plausible controls on these elements in an urban European setting.

Specifically, the study aimed to (a) quantify total concentrations and land-use-specific patterns of the selected metal(loid)s in urban, industrial and garden soils of Alcalá de Henares; (b) evaluate their degree of enrichment and contamination using local background values and pollution indices; (c) identify dominant source contributions through multivariate analyses and spatial distribution patterns and (d) characterise non-carcinogenic and carcinogenic human health risks associated with oral ingestion and inhalation of resuspended soils for different population groups (children, adults and workers), using current toxicological benchmarks where available.

In line with these objectives, we hypothesised that (i) industrial soils would show higher concentrations and enrichment of Co, Fe and Y due to historical and ongoing industrial activities; (ii) Ag and Sb would exhibit stronger enrichment in garden and urban park soils, reflecting inputs from urban consumer products and diffuse anthropogenic sources; (iii) Pt and Rh would be predominantly associated with vehicle emissions and traffic-affected areas and (iv) total soil concentrations of the monitored metal(loid)s would yield hazard quotients and cancer risks below conventional regulatory thresholds, although data gaps for Pt, Rh and Y would constrain full risk characterisation. By testing these hypotheses, the study provides new evidence on the geochemical behaviour and health implications of less-regulated metal(loid)s in urban topsoils and supports their inclusion in future monitoring and regulatory strategies.

## 2. Materials and Methods

### 2.1. Site Characteristics

Alcalá de Henares, a UNESCO World Heritage City located in central Spain, is approximately 31 km from Madrid along the A-2 motorway, 15 km from Madrid-Barajas international airport and 22 km from Guadalajara. As of 1 January 2022, its population stood at 196,888 [17], placing it among the most densely populated urban centres in the Comunidad de Madrid. According to OECD classifications, Alcalá de Henares is on the threshold of qualifying as a medium-sized city (200,001–500,000 inhabitants) [18]. Several industrial estates of varying activity levels are located on its outskirts.

Geologically, Alcalá de Henares is situated within the central basin of the Tajo River, which is composed primarily of Mesozoic, Paleogene and Neogene sediments. The region’s topography has been shaped by Quaternary geomorphological processes [19]. The study area lies on Quaternary alluvial terraces of the Henares River, overlying Miocene detrital clayey strata and adjacent to Miocene limestone escarpments known as the Cerros de Alcalá. Soils in this zone, classified within the Facies Madrid formation, are dominated by coarse-grained sands with variable clay content, ranging from loamy sands to coarse sands, and typically exhibit moderate to low permeability and near-neutral pH. These soil and substrate characteristics may influence both the natural background levels and the mobility of metal(loid)s. Alcalá de Henares experiences a continentalised Mediterranean climate, characterised by hot, dry summers and cold, wet winters.

**Figure 1:**
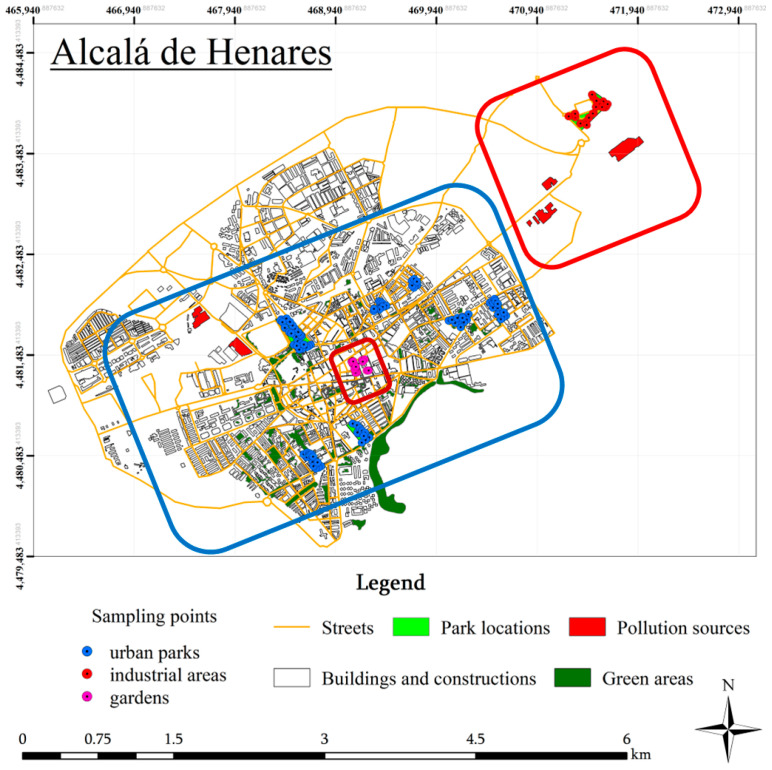
Study area and land-use-stratified sampling sites in Alcalá de Henares. Sampling points are classified as urban parks (blue), industrial areas (red), and gardens (magenta). The corresponding sampling zones are outlined using the same colour scheme.

The main plausible anthropogenic inputs of the monitored metal(loid)s in Alcalá de Henares include (i) traffic-related deposition, particularly non-exhaust emissions and resuspension (relevant to Sb and, for PGEs, catalyst-related inputs); (ii) industrial activities associated with peripheral industrial estates and legacy deposition; (iii) construction and urban materials, including demolition debris, fill and associated dust and (iv) managed-soil inputs in gardens and landscaped areas (e.g., imported soil and organic amendments), which can generate localised enrichment—particularly for Ag in intensively managed soils. In addition, the municipality has a long-standing history of construction-related and ceramic/brick manufacturing activities, which may contribute to legacy signatures for elements with low environmental mobility; these sectoral considerations are discussed further in Section 3.4 in the context of the observed element-specific patterns. Spatially, areas of elevated concentrations tend to occur near industrial land use polygons and major transport corridors (Figure 1), consistent with the source categories outlined above; however, definitive source attribution would require targeted source sampling and/or additional tracers.

### 2.2. Sampling

A total of 137 surface soil samples, representing the 0 to 3 cm layer, were obtained at random using a stainless steel manual auger during July 2001. This shallow surface layer was selected because it concentrates recent atmospheric deposition and is the soil horizon most frequently contacted by children and adults during recreational and gardening activities, making it particularly relevant for human exposure assessment. The sampling locations included urban parks (*n* = 97) and industrial (*n* = 22) and garden (*n* = 18) areas throughout Alcalá de Henares, as illustrated in Figure 1. In this study, urban parks refer to public recreational green spaces managed by the municipality (e.g., lawns, tree/shrub areas and landscaped park soils) that are primarily influenced by atmospheric deposition and routine maintenance. By contrast, gardens refer to intensively managed planted areas (including ornamental and/or allotment-style soils) where soil disturbance is more frequent and where additions of imported soil, compost, organic amendments or related inputs are more likely. Gardens may therefore also introduce a potential soil-to-produce pathway, reinforcing the value of treating them separately from passively used municipal parks. These areas may also involve more direct resident contact through gardening activities, which is relevant for interpreting localised enrichment patterns (e.g., Ag). Details on the full sampling protocol are available in Peña-Fernández et al. [20]. Although samples were collected in 2001, they were preserved under standard protocols and were processed and analysed recently using current instrumentation. Given that most of the monitored metal(loid)s are environmentally persistent, their total concentrations remain valid indicators of historical contamination and long-term anthropogenic accumulation trends. These samples thus provide a valuable baseline for assessing historical pollution patterns and support future comparative assessments of temporal variation.

After collection, soils were left to air-dry under ambient conditions, passed through a 2 mm mesh sieve and preserved in polyethylene containers [21]. Physicochemical properties and soil texture were analysed in accordance with Spanish official protocols [22]. Briefly, the pH and electrical conductivity (EC) were determined in a suspension using a 1:2.5 (*w*/*v*) ratio of topsoil and deionised water, and organic matter (OM) content was analysed following the Walkley–Black method. Soil texture was determined using a Bouyoucos densitometer [23].

### 2.3. Topsoil Analysis

Approximately 0.7 g of each air-dried, sieved topsoil sample was digested in 5 mL of 65% nitric acid (Suprapur, E. Merck, Darmstadt, Germany) using Teflon digestion vessels. Samples were left at ambient temperature for 8 h before heating at 96 °C for 12 h. The resulting solutions were cooled, filtered and diluted to a final volume of 25 mL with ultra-pure water (Milli-Q^®^ Direct 8, Merck Millipore, Darmstadt, Germany; resistivity 18.2 MΩcm) [21]. Procedural blanks were prepared and processed alongside the samples using the same reagents and digestion conditions.

Levels of Ag, Co, Fe, Mo, Pt, Rh, Sb, Se and Y were quantified by inductively coupled plasma mass spectrometry (ICP–MS; NexION 350D, PerkinElmer Inc., Waltham, MA, USA), operated with a collision cell using helium gas to minimise polyatomic interferences. Multi-element calibration standards were prepared daily in 2% HNO_3_, covering the concentration ranges expected in the digests. Calibration curves showed coefficients of determination (R^2^) ≥ 0.999 for all monitored elements. Internal standards were added online to correct for instrumental drift and matrix effects. Quality control was maintained by duplicate analyses and routine inclusion of a soil certified reference material (CRM059, loamy clay 2; Lot LRAA8361; Sigma-Aldrich (Merck), Taufkirchen (Munich), Germany) after every five samples. Certified concentrations (µg g^−1^) for selected analytes in CRM059 were as follows: Ag = 57.8 ± 1.16, Co = 98.8 ± 2.29, Fe = 5250 ± 146, Mo = 33.9 ± 0.79 and Se = 94.5 ± 3.07. An aqueous multi-element certified reference material (SPS-SW2; Spectrapure Standards AS, Oslo, Norway; batch 127) was also analysed as a control solution, with certified concentrations (ng mL^−1^ at 20 °C) of Co = 10 ± 0.05, Fe = 100 ± 1, Mo = 50 ± 0.3, Se = 10 ± 0.05 and Y = 2.50 ± 0.02. Measurements were performed in triplicate and quantified using the most abundant, least interfered isotope available for each element [24]. Analytical precision, expressed as relative standard deviation (RSD), typically ranged from 5–10%, and average recovery rates for the CRM were between 91% and 99%.

Method limits of detection (LoD) were estimated as three times the standard deviation of procedural blanks; for Se, the LoD was 0.327 mg kg^−1^, and all samples were below this value, so Se was not considered in the subsequent statistical or risk analyses.

Data for this article is available at Open Science Framework (OSF) at https://osf.io/8wsjk/?view_only=8012ed923ba9422298a6a772a06070df (accessed on 15 January 2026).

### 2.4. Statistical Analysis and Geochemical Mapping

Statistical methods appropriate for censored data (non-detects; i.e., data below the limit of detection, LoD) were applied, following recommendations by Helsel [25] and Shoari and Dubé [26]. These authors note that substituting one-half the detection limit for non-detects allows imprecise values to disproportionately influence total estimates, potentially compromising risk characterisation. Therefore, this procedure was employed: (a) for <50% censoring, use the Kaplan–Meier method; (b) for 50–80% censoring, apply robust regression on order statistics for sample sizes lower than 50 and maximum likelihood estimation for sample sizes higher than 50 samples; and (c) for >80% censoring, report high sample percentiles. These approaches were implemented using the ‘NADA’ package [27] in the R statistical environment, version 4.4.3 [28].

To evaluate inter-area differences in elemental concentrations, appropriate multiple comparison tests were applied based on the distributional properties of the data. For censored variables (i.e., those including values below the limit of detection), the Peto–Peto one-factor test was employed. For uncensored variables (i.e., all values above the limit of detection), the choice of test depended on the results of normality and homoscedasticity assessments. When both assumptions were met (normality and equal variances), Duncan’s new multiple range test was used. If normality was not rejected (assessed via the Shapiro–Wilk test) but homoscedasticity was rejected (assessed using the Fligner–Killeen test), pairwise Welch’s *t*-tests were conducted. If normality was rejected, pairwise Wilcoxon’s rank sum tests were used instead. Where applicable, *p*-values from multiple comparisons were adjusted using the Benjamini–Hochberg false discovery rate procedure. Statistical significance was determined at the *α* = 0.05 level. Principal component analysis (PCA) was applied to explore possible associations between elements and land use categories and to infer potential sources of the monitored metal(loid)s. Prior to PCA, variables were centred and standardised (z-scores) to minimise scale effects. Components with eigenvalues greater than 1 were retained following Kaiser’s criterion, and a varimax rotation was applied to facilitate interpretation of loadings [29]. In addition, PCA score-loading biplots (PC1 vs. PC2) were produced to visualise the relationships between elements and sampling sites by land use.

Geochemical mapping was used to reveal “hotspots”. Growing-dot maps were generated to visualise the spatial distribution of topsoil samples and highlight zones of potential element accumulation [30]. Mapping and spatial analysis were performed using the ArcGIS 10.1 software following established methodologies. Increasing colour darkness is proportional to the enrichment factor of each monitored element.

### 2.5. Pollution Index

Pollution indices (PIs) were calculated to assess the contamination levels of each monitored element across the main study area using the following equation [31]:

*PI* = *C_i_*/*C_b_*

In the above, *PI* is the evaluation score corresponding to each element/area, *C_i_* is the measured concentration of each element in the topsoils and *C_b_* is the geochemical background concentration of each element. For all the elements monitored, background values for Ag (0.07562), Co (5.602), Mo (0.3489) and Sb (0.2882; all in mg kg^−1^) were extracted from the specific geological unit in which Alcalá is located, based on the published guidelines of the *Instituto Geológico y Minero de España* [32].

### 2.6. Enrichment Factor

Enrichment factors (EFs) were calculated to quantitatively assess the anthropogenic contribution of each monitored element in topsoils from the main study areas [33]. *EF* was calculated for elements with known local background concentrations—namely Ag, Co, Mo and Sb [32]—using the equation described by Hu et al. [34]:

*EF* = (*C_i_*/*C_Mn_*)/(*B_i_*/*B_Mn_*),

In the above, *EF* is the standardisation of concentration of a measured element in topsoil/area with respect to the local background values and a sample reference metal, *C_i_* is the measured concentration of each element in the topsoil sample, *C_Mn_* is the concentration of manganese (Mn) in the same sample and *B_i_* and *B_Mn_* are the geochemical background concentration of each monitored element and Mn, respectively. Because common proxy elements (Al, Fe, Ti, Si, Sr, K, etc.) were not monitored in the guideline published by the *Instituto Geológico y Minero de España* [32], Mn was selected as the reference element for *EF* calculations. Mn values were extracted from Peña Fernández [35], and the element was chosen due to its low variability and lack of statistically significant differences between the three study areas. We acknowledge that Mn is not a fully conservative normaliser and may be influenced by paedogenic processes and, in some urban settings, anthropogenic inputs. Mn was nevertheless retained here because locally defined background values were available for Mn and for the target elements, enabling internally consistent *EF* comparisons across land uses; accordingly, the resulting *EF* values should be interpreted as semi-quantitative indicators rather than definitive source apportionment. *EF* values were categorised into six different categories [36,37].

### 2.7. Human Exposure and Health Risk Assessment

Human health risks were evaluated by estimating exposure to the total concentrations of each element in topsoils, following the methodology established by the US EPA [38,39,40] and consistent with the Technical Guide of Royal Decree 9/2005 [15,41]. The assessment considered two exposure pathways (incidental oral ingestion of soil and inhalation of soil-derived particles) for three population groups: children, adults and industrial workers (adults in the monitored industrial area of Alcalá).

Chronic daily intake (CDI) via incidental soil ingestion was estimated using the standard US EPA equations and default parameter values for ingestion rate, exposure frequency and duration, body weight and averaging time recommended in the RAGS guidance and the RAIS database [42,43]. Children were assigned higher soil ingestion rates and lower body weight, reflecting their greater vulnerability to incidental intake, while adult and worker scenarios used the corresponding adult defaults. For inhalation exposure, airborne concentrations of resuspended soil particles (C_air_) were derived from soil concentrations assuming a soil-to-air transfer based on the resuspension approach originally proposed by Hawley [44]. The resulting exposure concentration (EC) was then calculated using the US EPA inhalation dosimetry framework [40]. Ambient PM_10_ levels from the regional air-quality monitoring network in Alcalá during the sampling year were used as the basis for particle concentrations [45]. The full set of exposure equations and parameter definitions has been described in detail in the cited guidance documents and previous applications of this methodology [41] and is therefore not reproduced here.

Non-carcinogenic risks for each population group (children, adults and workers) were characterised using oral and inhalation hazard quotients (HQs), obtained by dividing the estimated exposure dose or concentration by the corresponding oral reference dose (RfDo) or inhalation reference concentration (RfCi). The RfDo and RfCi values applied in this study were taken from the US EPA Regional Screening Level (RSL) tables [46] and are reported together with the calculated HQ values in Section 3.5. Inhalation carcinogenic risk was evaluated only for cobalt, using an inhalation unit risk (IUR) of 9.0 × 10^−3^ (µg m^−3^)^−1^ obtained from the same US EPA RSL tables [46]. Health risks were interpreted according to US EPA criteria, considering HQ < 1 as indicative of negligible non-carcinogenic risk and acceptable lifetime cancer risks below 1 × 10^−6^ [39].

## 3. Results and Discussion

### 3.1. Characterisation of the Topsoils Collected in Alcalá de Henares

The physicochemical properties and texture of the topsoils monitored in the three main areas of Alcalá are summarised in Table S1 (Supplementary Files). The soils investigated demonstrated moderately basic characteristics, with pH values ranging from 6.89 to 9.06. Both pH and EC values were significantly higher in the topsoils collected from the industrial area. In contrast, organic matter content was significantly higher in the garden area, ranging from 0.23% to 8.60%. Spanish Mediterranean soils are typically characterised by high levels of pH and a low organic matter content [47]. The observed pH and electrical conductivity values were comparable to or lower than those reported in urban soils from Madrid and Sevilla [48,49]. In contrast, OM content in Alcalá’s soils was notably lower than in these comparative studies, except for samples collected in garden areas. Nevertheless, OM content was higher than the average reported in urban topsoils from Vigo (29,600  ±  12,900 mg/kg; equivalent to 0.0296%; [50]). Urban soils often display higher pH, OM and EC levels compared to rural or natural soils. These differences are driven by both direct and indirect contributions from construction debris and vehicular emissions, which enrich soils in metal(loid)s [51].

Based on standard classification methodologies [52], Alcalá’s soils are categorised as loam (urban and garden areas) and silt loam (industrial), textures typical of public park soils, which influence pollutant mobility and bioavailability through their physical and chemical properties. Overall, urban and garden soils exhibited similar sand and silt contents, whereas industrial soils showed significantly lower sand and higher clay percentages.

### 3.2. Elemental Concentrations and Pollution Indices in Topsoils Collected in Alcalá de Henares

Table 1, Table 2 and Table 3 provide descriptive statistics and regulatory benchmarks for Ag, Co, Fe, Mo, Pt, Rh, Sb, Se and Y across the three land use types (urban, industrial and garden). These include measured concentrations, local geochemical backgrounds, variability (CV) and pollution indices (PIs). Generic reference values/levels (GRVs/GRLs), established under Royal Decree 9/2005 for distinct land uses [15], are reported as screening thresholds. In addition, regional Spanish guidance values are included only as contextual screening comparators and are not intended to imply current regulatory exceedance or enforcement. Where Comunidad de Madrid guidance values were unavailable for a given element (e.g., Fe), we report the published value from Aragón as a pragmatic Spanish reference point and flag this explicitly in the relevant table. Table S2 compares concentrations across the land use categories.

Se was not detected in any of the samples analysed, suggesting that its presence was below the detection limit of the ICP-MS (0.327 mg/kg). This is consistent with the average crustal abundance of Se, reported to range between 0.05 and 0.09 mg/kg [53]. However, this detection limit is lower than the concentration reported in urban gardens from Madrid (0.73 mg kg^−1^), although it is higher than the regional natural median background level (0.18 mg kg^−1^) for Madrid soils [54]. Therefore, despite its non-detection, future monitoring studies should include Se, as elevated doses of this non-metal may enhance the carcinogenic potential of arsenic (As). Notably, our group has previously reported an increased risk of cancer following oral exposure to total As in these same topsoils [20]. In addition, Moreno Rodriguez et al. [55] reported Se concentrations ranging from 0.20 to 4.38 mg/kg in soils near Madrid, exceeding the 0.5 mg/kg threshold commonly cited to identify soils as potentially toxic for this metalloid.

Mo (92.8%), Sb (91.8%) and Rh (78.4%) presented high levels of censored data, suggesting minimal contamination in Alcalá’s urban topsoils. In contrast, Fe and Y were detected in all samples, as was Co, which exhibited only 1.03% censored data, indicating a homogenous distribution of these three metals across the monitored urban area. This trend was also observed in the garden and industrial areas, which is supported by their low CVs [56]. This is consistent with the anthropogenic usage of these elements and with previously reported data on their abundances in the Earth’s crust and urban soils, as compiled by Alekseenko and Alekseenko [57]: 46,500 and 22,300 (Fe) > 20 and 23.4 (Y) > 18 and 14.1 (Co) > 1.1 and 2.4 (Mo) > 0.07 and 0.37 (Ag) > 0.5 and 1 (Sb), all in mg/kg, respectively.

Relatively little is known about the abundance of platinum group elements [PGEs; platinum (Pt), palladium (Pd) and rhodium (Rh)] in the Earth’s crust, as detecting them is challenging, with most of their mass believed to reside in the core [58]. These authors estimated PGE concentration in the continental crust as 1.5, 1.4 and 0.35 ng/g, respectively. Park et al. [59], analysing loess samples from Argentina, China and Europe, proposed lower values of 0.60, 0.53 and 0.018 ng/g, respectively. More recently, Mitra and Sen [60] reported PGE abundances of 0.51, 0.52 and 0.6 ng/g, based on data from multiple studies.

The primary anthropogenic source of PGE contamination is attributed to automobile catalytic converters, which were introduced in Europe during the 1980s. This likely explains the higher proportion of censored data for Rh, in combination with its lower measured concentrations compared to Pt in Alcalá’s urban topsoils. Supporting this, a study by Moldovan et al. [61] on gasoline and diesel vehicles in Madrid, equipped with catalytic converters manufactured in 1998, revealed higher emissions of Pt (610 g/year) relative to Rh (190 g/year).

Except for Ag and Mo, the concentrations detected in Alcalá’s soils were lower than the median baseline concentrations reported for European topsoils in the Forum of European Geological Surveys (FOREGS) dataset (all in mg/kg; [62]): Co (7), Fe (19,600), Sb (0.60) and Y (21). The Ag concentrations observed in garden soils exceeded the FOREGS baseline for European soils (0.27 mg/kg; [62]), suggesting an anthropogenic contribution of silver in these areas. Notably, the P95 value for Ag in urban soils (0.611 mg/kg) was nearly nine times higher than the median (0.068 mg/kg), indicating a strongly right-skewed distribution. This statistical pattern is consistent with localised anthropogenic inputs, possibly from discrete urban sources such as treated wastewater, surface runoff, or consumer product residues. A similar trend was found for Mo in specific locations within urban and industrial areas, where some samples exhibited Mo levels above its reported baseline (0.62 mg/kg). To date, FOREGS has not provided baseline values for Se, Pt or Rh.

The concentrations of Ag, Co, Mo, Sb and Se did not exceed the GVRs established for urban, industrial and garden topsoils in the Madrid region ([16,63]; Table 1, Table 2 and Table 3). Likewise, Fe levels remained below the GVR set for soils in Aragón (Spain; [64]), except in garden topsoils (10,202.33 vs. 3750 mg/kg). This exceedance may reflect intensive anthropogenic disturbance in soils from garden areas such as Plaza Cervantes, located in the city centre of Alcalá. Notably, no GVRs have been established for Pt, Rh or Y in soils in any Spanish region.

In general, the monitored elements exhibited lower concentrations than those reported in urban soils from Madrid (0.068 vs. 3.30 Ag; 1.577 vs. 6.11 Co; 5405.05 vs. 22,100 Fe; 4.982 vs. 23 Y; all median values in mg/kg) [8], suggesting that Alcalá may be less contaminated with these elements compared to the Spanish capital. The concentrations were also lower or comparable to the natural background levels reported by the same authors during a 1990 sampling campaign covering 1200 km^2^ east of Madrid [65]: <0.5, 7, 17,800 and 14 mg/kg, respectively. Furthermore, the ranges observed in this study were below those reported in topsoils for Co (1.07–37.59), Fe (32,000–546,000), Mo (0.08–2.19) and Se (0.11–2.63) in the *Geochemical Atlas of Spain* [66,67].

**Figure 2:**
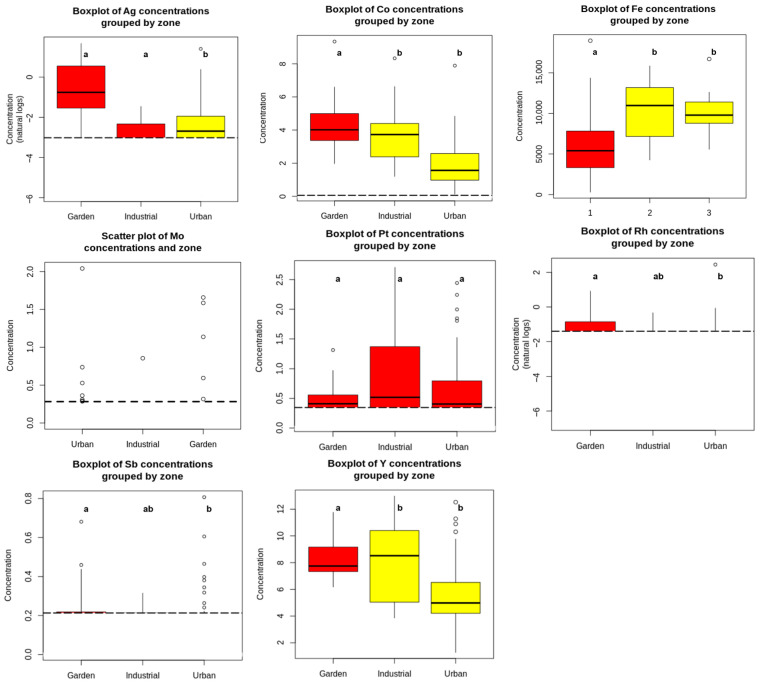
Levels of metal(loid)s in topsoils (*n* = 137) monitored in each main area in Alcalá de Henares. Box-and-whisker plots of trace element concentrations. Pt and Rh concentrations are shown in ng/g; all other elements are in mg/kg. The central line indicates the median; boxes represent the interquartile range (25th–75th percentiles); whiskers extend to values within 1.5 times the interquartile range. Points beyond the whiskers are outliers, while ‘extremeliers’ indicate values exceeding three times the interquartile range. Mean concentrations (arithmetic mean ± SD) with different letters denote statistically significant differences (*p*-value <  0.05). Colours represent these statistically distinct groups.

All elements except Mo and Pt (*p*-value = 0.195) showed statistically significant differences by area (Table S2), although the distributional differences were relatively modest (Figure 2). Specifically, Co, Fe, Pt, Rh and Y exhibited significantly higher concentrations in the industrial and garden areas, whereas Ag and Sb were significantly elevated in the urban area. Several extreme values were also observed for Ag and Rh, particularly in urban and garden soils. These data points, which lie beyond three times the interquartile range, likely reflect localised anthropogenic inputs, such as traffic hotspots, biosolids application or legacy contamination, which disproportionately influence area-wide concentrations. Their presence underscores the spatial heterogeneity of urban pollution and reinforces the need for targeted, site-specific risk management strategies.

Although the differences in Mo concentrations were not statistically significant, likely due to the high percentage of censored values, slightly elevated levels were observed in industrial topsoils, with the highest presence recorded in garden soils. These distributions align with the anthropogenic uses of the monitored elements and Alcala’s industrial activity and vehicle emissions. The use of Ag and silver nanoparticles in solar panels, water purification systems and medical applications, including consumer goods with antimicrobial properties, may explain the slightly higher Ag concentrations in urban soils compared to the industrial area (0.068 vs. 0.041 mg/kg). Additionally, the applications of biosolids from wastewater treatment and Ag-containing pesticides in urban landscapes are recognised sources of silver, which could explain the significantly elevated Ag levels in garden soils (0.439 vs. 0.068 and 0.041 mg/kg; *p*-value < 0.0001) [68]. As previously noted, an anthropogenic impact on Ag was particularly evident in garden areas of Alcalá. Generally, the concentration of Ag in surface soils ranges from <0.01 to 5 mg/kg, with most reported values below 1 mg/kg [69]. Our findings are consistent with studies showing that Ag levels in industrially impacted soils may be 3–20 times higher than the background concentrations [70].

The presence of Co in urban topsoils is typically attributed to natural processes, as reported in recent studies [9,71], which may also apply to Alcalá when considering the *EF* values presented in Table S3 and discussed further below. However, higher concentrations of Co are often found in locations with intense human activity and traffic density [72], which could explain the values observed in this study. Cobalt is used in superalloys, special steels and rechargeable batteries found in electronic devices such as mobile phones and laptops [72]. The Co concentration detected in Alcalá’s urban topsoils was considerably lower than the arithmetic mean reported in urban topsoils from Constantí (1.88 vs. 5.48 mg/kg), a city hosting a hazardous waste incinerator in Catalonia, Spain [73]. Similarly, it was much lower than the concentrations reported for surface horizons in urban/industrial soils in eastern Spain (11.0 mg/kg) [74]. The Co levels observed in Alcalá were also within or below the geochemical baseline range (1.2–35.5 mg/kg) reported for uncontaminated soils by these authors.

Molybdenum is considered an emerging environmental contaminant, though it has been assessed in only a limited number of topsoil studies [75]. Its sources and geochemical behaviour in urban environments remain poorly characterised. Mo in soils has been associated with mining and industrial applications, particularly in the production of optoelectronic materials and semiconductors, high-strength steel alloys, catalysts, corrosion inhibitors, ceramics, vacuum tubes, lubricants and pigments. These potential sources might explain the higher concentration of Mo detected in the soils monitored in the industrial areas in Alcalá but cannot explain the higher distribution detected in the soils collected in the gardens. Despite low detection frequency, the relatively high *EF* in some garden and industrial soils described later may suggest specific point-source inputs such as industrial catalysts or agricultural applications. In comparison, Mo levels in the urban soils of Yerevan, Armenia, home to a Mo concentrate processing plant, were reported at 4.70 mg/kg [76], which is substantially higher than the concentrations detected in Alcalá. Furthermore, Mo levels in our study were below the median concentration reported for European grazing land soils in the “Geochemical Mapping of Agricultural and Grazing Land Soil of Europe (GEMAS)” project (0.42 mg/kg; [77]). Overall, our results suggest minimal Mo pollution in the topsoils of Alcalá.

The significantly higher levels of Sb in urban areas (0.352 vs. 0.292 and 0.124; Table S2) may be attributed to its use in vehicle brake linings and tires, flame-retardant plastics, polyethylene terephthalate bottles and the manufacture of paints and batteries [78,79]. Sb also plays a key role in semiconductor technologies and manufacturing industries [79], which may further explain the distribution pattern observed in the study area. Due to its extensive industrial applications, Sb has been classified as an emerging or priority pollutant in both Europe and the US [9,78]. Nonetheless, the presence of Sb was more evident in urban garden topsoils from Alcalá, although concentrations were much lower than those reported in urban gardens in Madrid (0.124 vs. 0.90 mg/kg; [54]). Similarly, Vilavert et al. [73] reported an arithmetic mean Sb concentration of 0.15 mg/kg in urban topsoils from Constantí (Spain). However, in our case, we were unable to report an arithmetic mean due to the high proportion of censored data in urban areas (91.8%), suggesting limited Sb contamination in Alcalá.

The presence and distribution of Pt and Rh in urban soils were initially reported in Peña-Fernández et al. [80]; however, in that study, censored values were substituted using a fraction of the detection limit. This traditional substitution method, although common in environmental chemistry, can obscure data patterns and distort statistical inferences, potentially compromising the detection of significant differences, correlations and relationships [25,81]. Neglecting censored values primarily affects the lower end of the distribution, making summary statistics such as means, medians, percentiles and standard deviations unreliable [82]. To address this, we have re-processed the dataset using statistically robust methods recommended by Helsel [25], which have led to lower Pt and Rh concentrations in urban topsoils in the present study compared to our earlier work (0.404 vs. 0.488 ng/g for Pt; 0.084 vs. 0.110 ng/g for Rh). The greater reduction observed for Rh likely reflects the higher proportion of censored values for this element. Regarding spatial distribution, Pt levels did not differ significantly across land use categories, whereas Rh concentrations were significantly higher in garden soils compared to urban soils (0.168 vs. 0.084 ng/g; *p*-value < 0.05; Table S2).

Rare earth elements (REEs) are a group of metals whose use has increased significantly in recent decades. Yttrium, which is considered an REE due to its chemical similarity to the lanthanides, is primarily used in LED televisions, computers and mobile phones and can also be found in cameras and electric vehicle batteries [83]. Y is utilised individually or in combination with other REEs to produce phosphors for cathode ray tubes and flat panel displays, as well as fluorescent and light-emitting diode (LED) lighting [84]. Because locally defined background values were not available for Y in the cited regional unit, Y enrichment was evaluated separately using a crust-normalised approach (Fe-normalised upper continental crust ratio) and is therefore not directly comparable to the Mn-normalised *EF* values reported for Ag, Co, Mo and Sb. The enrichment factor for Y was calculated relative to its average concentration in the Earth’s upper continental crust (UCC), using data from Taylor and McLennan [85] and the following equation: EF_earth crust_ = [Y/Fe]_sample_/[Y/Fe]_upper Earth crust_. As data from scandium is unavailable in this study, Fe was selected as the normalisation element, as it has been shown to be a reliable proxy for this purpose [86]. The calculated *EF* values were 1.50 (urban), 1.25 (industrial) and 1.27 (garden), indicating that Y is present at natural background levels, with its accumulation likely resulting from persistent but limited anthropogenic deposition [87]. However, the concentration of Y was significantly lower in the urban area compared to the other areas (4.982 vs. 8.518 and 7.745 mg/kg). Y concentrations in Alcalá were also lower than those reported for topsoils from urban parks and industrial zones in Chelyabinsk, Russia, a highly industrialised city (9.6 and 19.1 mg/kg, respectively; [88]), and substantially lower than the median reported for Lamia City, Greece (12.8 mg/kg; [89]). These findings suggest low Y contamination across Alcalá de Henares. However, it is important to note that Y is often associated with a zirconium matrix, which may not be fully dissolved by wet digestion methods such as that followed in this work [90]. Therefore, a proportion of the variation observed between studies, estimated at around 10 to 20 percent, may be attributed to differences in analytical recovery and methodology.

Examination of the *PI* values for Ag (mean: 2.46; range: 0.65–53.55; Table 1, Table 2 and Table 3) in urban soils and Ag in garden soils (mean: 16.2; range: 1.13–71.36), as well as Mo in industrial (2.45; single value) and garden soils (mean: 1.04; range: 0.91–4.75), when considered alongside CVs exceeding 100% (239.48 and 115.85 for Ag, 393.36 and 106.35% for Mo), strongly suggests an anthropogenic origin for these elements [91]. CVs greater than 100% also reflect high spatial variability, as observed for Ag and Rh in urban areas and for Ag, Mo, Rh and Sb in industrial areas [56]. The high coefficients of variation and right-skewed distributions for Ag, Mo and Rh—particularly the elevated P95 values—further support the interpretation of point-source anthropogenic contributions in certain hotspots. Furthermore, based on *PI* > 1 criteria, Ag (49.5%), in urban areas; Ag (27.3%), Co (9.1%), and Sb (9.1%) in industrial areas and Ag (94.4%), Co (16.7%), Mo (22.2%), and Sb (22.2%) in garden areas may be associated with industrial activity, vehicle emissions or other anthropogenic sources. Despite these findings, the relatively low mean, minimum and maximum *PI* values for Co, Mo and Sb across all areas suggest shorter contamination periods and lower accumulation rates for these elements.

### 3.3. Elemental Enrichment, PCA and Potential Sources of Metal(loid)s in Topsoils Collected in Alcalá de Henares

The mean, maximum and minimum *EF* values for Ag, Co, Mo and Sb are reported in Table S3 for each land use area, as BVs have so far only been defined for these elements. The mean *EF* values of Ag (3.18) in urban soils, Co (1.11, 1.20, 1.45) in urban, industrial and garden soils and Mo (1.47, 1.07) in industrial and garden soils were close to the threshold of 1.5, supporting the presence of anthropogenic contributions, as previously noted [9]. The urbanisation and high population density in Alcalá may account for the distribution pattern of Sb, whereas the moderate-to-high enrichment levels of all four elements in garden soils are consistent with common anthropogenic inputs typical of these environments. In particular, the very high enrichment factor of Ag in gardens (mean: 14.076; max: 69.3) and the finding that 16.7% of garden samples exceeded *EF* > 40 and 27.8% had *EF* > 10 provide compelling evidence of localised anthropogenic input, possibly from consumer product degradation or biosolids. Given this degree of enrichment, further investigation into the potential for bioaccumulation of Ag in edible urban produce or incidental exposure in recreational settings is warranted.

Garden soils represent a sensitive land use because contact frequency and soil disturbance can be higher than in passively used parks, increasing opportunities for incidental ingestion and inhalation of resuspended particles during gardening. In addition, gardens may function as food-growing environments, creating a potential secondary pathway via consumption of home-grown produce. While the present work evaluates total concentrations as a baseline indicator, risk characterisation in gardens would benefit from follow-up measurements of metal(loid) bioaccessibility and soil-to-crop transfer, particularly for Ag given its pronounced enrichment and hotspot behaviour in this land use. Recent citywide and exposure-oriented studies of urban gardens similarly emphasise that garden-specific practices and produce pathways can be relevant to interpreting soil contamination patterns and potential health implications [92].

The pronounced spatial heterogeneity and hotspot behaviour observed for Ag (especially in gardens) and, to a lesser extent, Mo (high censoring but locally elevated values) are consistent with point-scale anthropogenic inputs superimposed on a largely geogenic matrix. However, source attribution from concentration patterns and multivariate structure alone must remain evidence-proportionate. For Ag, a plausible pathway in intensively managed soils is the use of compost/biosolids or related organic amendments, since wastewater treatment can concentrate Ag in sewage sludge, and biosolid application has been shown to increase soil Ag over time; diffuse urban inputs and consumer product residues may also contribute [93]. For Mo, traffic-related activity is a plausible contributor because non-exhaust emissions and brake-related materials contain a complex assemblage of mineral phases and trace metals that can be deposited to adjacent soils, in addition to any local industrial influences [94]. Definitive apportionment would require complementary source sampling and/or tracer approaches (e.g., isotopic or mineralogical/speciation evidence), which were beyond the scope of the present baseline study.

**Figure 3:**
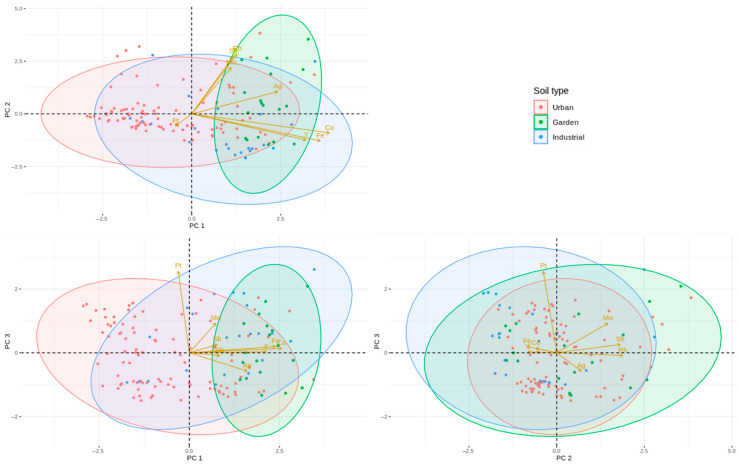
Principal component analysis (PCA) biplot of metal(loid)s in Alcalá de Henares topsoils by soil type. Scores are coloured according to soil type (urban, garden, industrial), and loadings for each element (Ag, Co, Fe, Mo, Pt, Rh, Sb, Y) are represented as vectors. PC1 and PC2 explain 35.2% and 18.7% of the total variance, respectively. Co, Fe and Y load strongly on PC1, suggesting a predominantly geogenic association, whereas Rh, Sb and Mo are aligned with PC2, indicating anthropogenic contributions. Pt contributes mainly to PC3, consistent with a distinct source related to traffic and other localised inputs of platinum group elements.

To explore the potential origins of metal(loid) concentrations, PCA was applied. PCA is used here as an exploratory multivariate tool to summarise co-variation patterns among elements and land uses. PCA loadings therefore identify shared variance and do not, by themselves, demonstrate causation or uniquely confirm anthropogenic versus geogenic sources; accordingly, the following interpretations are presented as plausible hypotheses consistent with the spatial/land use patterns. Robust source apportionment would require complementary evidence (e.g., mineralogical/speciation data, isotopic tracers or measured source profiles/receptor modelling), which was outside the scope of this baseline study [95]. Three principal components with eigenvalues greater than 1 were identified, together explaining 66.4% of the total variance (Table S4; Figure 3). PC1 had an eigenvalue of 2.75 and accounted for 34.3% of the variance; it showed very strong positive loadings for Co (0.93), Fe (0.87) and Y (0.77) and a moderate loading for Ag (0.58), suggesting a predominantly geogenic association for Co, Fe and Y, with Ag partly following this pattern. PC2 (eigenvalue 1.52; 19.0% of the variance) was characterised by moderate-to-strong positive loadings for Rh (0.68), Sb (0.66) and Mo (0.53), indicating a common anthropogenic influence on these elements. PC3 (eigenvalue 1.04; 13.0% of the variance) was dominated by Pt (loading 0.93), with a smaller contribution from Mo (0.34), consistent with a distinct source largely related to traffic emissions and other localised inputs of platinum group elements [96]. Emissions of Pt and other platinum group elements from catalytic converters are known to vary with vehicle speed, catalyst design and age, fuel type and driving conditions [97]. In the PC1–PC2 biplot (Figure 3), Co, Fe and Y cluster along PC1, whereas Rh, Sb and Mo align with PC2, illustrating the contrast between mainly geogenic and anthropogenic components. Because Pt contributes primarily to PC3, it is only weakly expressed in the PC1–PC2 plane. Therefore, the PC1–PC2 biplot should not be interpreted as fully representing PGE behaviour, and inferences regarding Pt are supported primarily by PC3 loadings (Table S4) and spatial patterns rather than the PC1–PC2 plane alone. In addition, PCA identifies co-variation structures and is not, by itself, proof of source causation; source interpretations are therefore presented as plausible hypotheses consistent with land use and mapping results. Iron is typically associated with a lithogenic background due to its abundance in parent materials and its key role in paedogenesis [9], and Co and Y often follow similar patterns in urban and semi-natural soils, supporting their predominantly natural origin in this setting.

Table S5 summarises the PCA results for the elements monitored exclusively in the urban area, revealing a broadly similar pattern to the full dataset. Three principal components accounted for 63.6% of the total variance. Silver (Ag), Co, Fe and Y exhibited moderate-to-strong positive loadings on the first component, whereas Rh and Sb were primarily associated with the second component, and Mo loaded mainly onto the third. These distributions are consistent with anthropogenic influences on Mo and Rh in urban topsoils, superimposed on a largely geogenic background for Co, Fe and Y.

### 3.4. Spatial Distribution and Correlations of Metal(loid)s in Topsoils Collected in Alcalá de Henares

Supplementary Table S6 summarises the evidence base (land use contrasts, *EF*/*PI*, PCA and spatial hotspots) supporting the element-specific, hypothesis-based interpretation of plausible sources/controls discussed in this section.

Geochemical maps are essential tools for visualising the spatial distribution and identifying the potential sources of environmental contaminants. The maps generated for Alcalá (Figures S1 and S2; Supplementary) revealed a similar concentration pattern for Ag, Co, Mo, Rh and Sb, with elevated concentrations (above the 75% percentile) observed in central urban parks and the monitored garden areas. In contrast, Fe, Pt and Y displayed moderate-to-high concentrations across all three land use areas, suggesting a more homogeneous spatial distribution. The geochemical maps also revealed pronounced spatial clustering of Co, Fe and Y in the industrial zones, supporting the interpretation that these elements are strongly associated with historical or ongoing industrial emissions.

Spatially, “hotspots” of the monitored metal(loid)s largely coincide with historical industrial zones and the current locations of pollution sources (Figure 1). Several factories and industries facilities, either currently operating or formerly active in Alcalá de Henares, are likely contributors to the observed distribution patterns. These include firms in information technology (e.g., Cerosyunos Ingeniería Informática SLL, Repuestos y Suministros del Henares SL); electronics (e.g., Suministros Electrónicos Alcalá SL, which supplies the Department of Electronics at the University of Alcalá and collaborates with multinational corporations such as Pirelli, Siemens, Osram and Zemper Alta Tecnología); photography (e.g., Estudio Foto Arte SL, Estudio Fotográfico Panta SL, Digifoto Alcalá SL); glass manufacturing (Saint Gobain Vetrotex SE, a fibreglass producer) and ceramics (e.g., Roca and Alcalagress). Alcalá de Henares has a long-standing history as one of the main ceramic-producing municipalities in the Comunidad de Madrid, particularly for construction bricks, an industry that began in the 1940s to meet the demands of Madrid’s urban expansion [98]. Although ceramics production has significantly declined, its historical emissions may still be reflected in the soil, especially for elements with low environmental mobility, which can persist in topsoils long after industrial activity has ceased.

The Pearson correlation coefficient matrices for metal(loid)s across land use types are presented in Figure S3, while the matrices evaluating correlations between metal(loid) concentrations and physicochemical properties or soil texture area are shown in Figure S4. In the urban area, positive correlations were found between most metal(loid) pairs, whereas Pt displayed significant negative correlations (*p*-value < 0.05) with Ag (*r* = −0.242), Co (*r* = −0.241) and Y (*r* = −0.216). Fe emerged as a particularly influential variable, likely reflecting its dominance as the major soil element in Alcalá’s urban parks. Strong and statistically positive correlations (all *p*-value < 0.01) were identified for the following element pairs: Fe-Co (*r* = 0.853), Fe-Y (*r* = 0.419), and Fe-Ag (*r* = 0.289). These findings are noteworthy given both the broad spatial coverage of Fe (Figure S1) and its status as the element detected at the highest concentration in the study (Table 1). Moreover, several authors have highlighted the key role of Fe in regulating the distribution and mobility of metal(loid)s in soils [99].

Regarding soil parameters (Figure S4), significant positive correlations were observed between OM and Fe (*r* = 0.335) and OM and Co (*r* = 0.375), as well as between EC and Sb (*r* = 0.368). In contrast, EC showed a significant negative correlation with Pt (*r* = −0.341); all correlations had *p*-values < 0.05. Interestingly, no significant correlations were identified between soil parameters and metal(loid) concentrations in the industrial or garden areas. This lack of correlation may be attributed to the smaller sample sizes collected in these areas and the greater degree of anthropogenic disturbance, which can obscure geochemical relationships. The absence of statistically significant correlations between pH and metal(loid) concentrations across all three areas aligns with previous studies, which suggests this may be due to heterogeneous contamination sources, variability in soil types and the influence of fertiliser use [99].

In contrast, significant correlations were observed between soil texture and several metal(loid)s in both the urban and industrial areas, although the associations varied by area and element. In urban soils, strong negative correlations were identified between sand content and both Fe (*r* = −0.432) and Co (*r* = −0.464), while strong positive correlations were found with clay content (*r* = 0.563 and 0.570, respectively). Meanwhile, in industrial soils, Rh was positively correlated with sand (*r* = 0.681) and negatively with silt (*r* = −0.681), and Sb exhibited a strong positive correlation with sand (*r* = 0.723) and a strong negative correlation with clay (*r* = −0.764). These patterns are consistent with previous research [100], which attributed such associations to the lithogenic nature of elements like Fe and Co and the anthropogenic sources of Rh and Sb. These findings are also in agreement with the PCA results, further supporting their reliability.

### 3.5. Risk Assessments

Non-carcinogenic risks from oral exposure were assessed for all monitored elements except Pt and Rh, for which oral reference doses (RfDo) have not yet been established by the US EPA [46]. The calculated chronic daily intake (CDI_oral_) and corresponding non-carcinogenic hazard quotients (HQ_oral_) are presented in Table 4, disaggregated by population group and sampling area. For yttrium, the provisional RfDo for yttrium chloride, proposed by TERA [101], was used to evaluate potential oral toxicity. Mo was excluded from the urban and industrial risk characterisation due to a high percentage of censored data, with detection rates below 10%.

Non-carcinogenic risks from inhalation exposure were characterised for Co, Mo and Sb, as inhalation reference concentrations (RfCi) are currently only available for these elements [46]. The calculated inhalation hazard quotients (HQ_inh_) for each monitored area are summarised in Table 5.

As expected, CDIs and HQs were highest for children in both urban and garden areas, reflecting their greater soil ingestion rates and lower body weight. However, for all elements and exposure pathways, HQ values for children, adults and workers remained well below 1 and did not exceed 0.2, indicating low estimated non-carcinogenic risks for elements with established toxicity benchmarks, even under the most conservative child scenario. Accordingly, the overall risk conclusions should be read as applying to the benchmarked elements, while Pt and Rh remain a recognised uncertainty that warrants future toxicological and exposure refinement. While Co and Fe are essential trace elements, exposure to elevated levels can be detrimental to human health. Although the characterised risks in this study were minimal, the growing use of elements such as Co, particularly in new vehicle technologies [9], underscores the need to include them in future environmental monitoring programmes.

The carcinogenic risk from inhalation exposure to Co was calculated at 1.05 × 10^−7^, 2.52 × 10^−7^ and 2.13 × 10^−7^ for adults in urban and garden areas and for workers in the industrial area, respectively. These results indicate a very low cancer risk, as all values were below the US EPA safety threshold of 1.0 × 10^−6^. Importantly, quantitative risk metrics (HQ/HI and cancer risk) can only be calculated for elements with authoritative toxicological benchmarks for the relevant exposure route(s) (e.g., RfD/RfC and, where applicable, inhalation unit risk). For Pt and Rh, widely accepted benchmark values remain unavailable for the pathways assessed, and for Y, benchmark coverage is limited and/or less harmonised; therefore, formal HQ/cancer risk calculations could not be derived for these elements. Consequently, the “very low risk” conclusion applies to the subset of elements with established benchmarks and should not be interpreted as evidence of absence of risk for Pt/Rh/Y.

While the samples represent conditions at the time of collection (2001), the long environmental half-lives and limited mobility of many monitored elements (e.g., Pt, Rh, Sb, Co) support the relevance of our findings in evaluating persistent anthropogenic contamination. Nevertheless, key drivers of inputs for some monitored elements have changed since 2001. In particular, Pt and Rh emissions are sensitive to the composition and age of the vehicle fleet, catalyst formulation and durability and traffic intensity; accordingly, present-day spatial patterns may differ from those captured in this historical snapshot [97,102]. Silver inputs may also have evolved due to changes in consumer product composition, the emergence of Ag-containing antimicrobial materials and shifts in urban waste and organic amendment practices [103,104]. For these reasons, our findings are best interpreted as documenting historical accumulation and land use contrasts in Alcalá de Henares and as providing a baseline against which contemporary resampling can test temporal trends [97,102,104]. Future studies should investigate the environmental fate of these elements in the soils, including how soil texture and physicochemical properties influence their mobility, distribution and persistence, to better understand the factors regulating soil contamination risks. Currently, there is limited evidence on the role of soil properties in controlling the mobility and bioavailability of certain metal(loid)s, such as Mo [75]. Expanding scientific knowledge on the behaviour of these contaminants in urban soils, as well as their potential effects on human and ecological health, is essential for promoting sustainable urban development and identifying effective contamination control strategies for improved soil management.

Overall, the observed land use contrasts and enrichment patterns are consistent with the broader framework of urban soil pollution, in which contaminant mixtures reflect (i) traffic-related non-exhaust inputs and resuspension, (ii) industrial/urban legacy deposition and localised hotspots and (iii) managed soil inputs (e.g., amendments in gardens), superimposed on a lithogenic background. In this context, the inclusion of less-regulated, technology-related elements (PGEs and Y) complements conventional urban metal assessments by highlighting emerging indicators of modern urban activities.

Beyond these environmental considerations, important toxicological evidence gaps remain for Pt, Rh and Y that directly constrain quantitative risk characterisation. In the absence of authoritative route-specific toxicity values for Pt and Rh, and given the limited harmonisation of benchmark values for Y across frameworks, formal HQ/CR calculations cannot be derived for these elements; therefore, “low risk” interpretations apply only to the subset of metal(loid)s with established benchmarks and do not exclude potential risks from Pt/Rh/Y. Available toxicological syntheses suggest that traffic-related airborne platinum group elements can trigger oxidative stress and pro-inflammatory responses, with effects strongly dependent on chemical form and bioaccessibility; notably, soluble Pt salts are established respiratory sensitisers in occupational settings [105], highlighting the importance of speciation for hazard characterisation. In parallel, recent reviews of rare earth elements, including Y, repeatedly implicate oxidative stress and inflammatory responses as plausible mechanisms across organ systems, although dose–response evidence remains insufficiently mature to support widely accepted soil exposure benchmarks [106], particularly for Rh, where inhalation toxicity data are scarce [105,107]. Notably, recent toxicological syntheses have highlighted Y as an REE of potential concern in comparative assessments, although toxicity rankings depend on endpoints, speciation and exposure conditions [106,108]. Accordingly, future research should prioritise speciation-aware assessments (including bioaccessibility) and targeted toxicological studies across relevant exposure concentrations to strengthen benchmark development and refine risk estimates.

## 4. Conclusions

Oral and inhalation exposures to Ag, Co, Fe, Mo, Sb and Y via soil ingestion and inhalation of resuspended particles in Alcalá de Henares were below established risk thresholds for all population groups, including children. For all elements and exposure pathways, hazard quotients did not exceed 0.2, and inhalation cancer risks for Co were ≤2.5 × 10^−7^, well below the conventional benchmark of 1 × 10^−6^. These results indicate low estimated non-carcinogenic and carcinogenic risks under the exposure scenarios considered for elements with established toxicity benchmarks, while risk characterisation for Pt and Rh remains qualitative due to benchmark gaps.

From a geochemical perspective, industrial soils exhibited the highest overall contamination levels, followed by garden soils, with urban park soils showing the lowest enrichment. Industrial areas were characterised by elevated Co, Fe and Y, reflecting a predominantly geogenic signature with superimposed industrial inputs, whereas garden soils displayed pronounced silver enrichment, with Ag pollution indices up to 71 and enrichment factors up to 69, about one sixth of samples (16.7%) exceeding *EF* > 40 and more than one quarter exceeding *EF* > 10. This pattern strongly suggests localised anthropogenic inputs, such as consumer product residues, biosolids or other urban sources. In contrast, Y enrichment factors were close to unity, indicating concentrations near natural background levels, despite its growing use in modern technologies.

Platinum group elements (Pt, Rh) were present at low concentrations but showed spatial patterns consistent with traffic-related emissions, as supported by PCA and spatial distribution analyses. However, the absence of oral reference doses and inhalation toxicity benchmarks for Pt and Rh, together with limited toxicological information for Y, constrains a full quantitative health risk characterisation for these elements. The long environmental persistence and low mobility of many of the monitored metal(loid)s also mean that the 2001 topsoil dataset provides a valuable baseline for evaluating historical contamination and for future temporal comparisons under evolving urban, industrial and regulatory conditions.

Overall, this study demonstrates that less-regulated, technology-related metal(loid)s can exhibit marked spatial heterogeneity and strong enrichment in specific urban land uses, particularly gardens, even where conventional regulatory thresholds are not exceeded. The findings emphasise the need to integrate platinum group elements and rare earths into urban soil monitoring programmes and to develop robust toxicity benchmarks and environmental quality standards for these contaminants. Such efforts are essential to strengthen evidence-based human health risk assessments, guide targeted remediation or management actions in sensitive land uses and support the broader objectives of European soil protection and sustainable urban planning.

## Figures and Tables

**Table 1:** Statistical summary of metal(loid)s in urban soils of Alcalá (mg kg^−1^), their background values (BVs), coefficients of variation (CVs), pollution indices (PIs) and Regulatory Guidance Values (RGVs; mg kg^−1^) (BOCM, Order 761/2007).

Element	LoD	*N*	% Cens	Arithmetic Mean	Geometric Mean	Median	Range	Interquartile Range	P95	CV (%)	BV	Pollution Index			*PI* > 1%	RGV
												Mean	Min.	Max.		
**Ag**	0.049	62	36.1	0.186 ± 0.446	0.072	0.068	0.049–4.049	<0.049–0.143	0.611	239.48	0.076	**2.46** ^c^	0.65	**53.55**	49.5	50
**Co**	0.059	96	1.03	1.880 ± 1.229	1.485	1.577	0.093–7.890	1.092–2.584	4.269	65.39	5.602	0.34	0.02	**1.41**	1.0	150
**Fe**	0.189	97	0.0	5796.86 ± 3654.33	4371.794	5405.05	315.60–18,956.23	3319.15–7818.30	11,496.66	63.04	/	/	/	/	/	10,000 ^d^
**Mo**	0.282	7	92.8	/	0.037	/	0.286–2.041	/	0.295	/	0.349	0.28	0.82	**5.85**	4.1	150
**Pt ^a^**	0.345	52	46.4	0.655 ± 0.482	0.386	0.404	0.355–2.443	<0.345–0.795	1.805	73.58	/	/	/	/	/	/
**Rh ^a^**	0.246	21	78.4	0.199 ± 0.425	0.085	0.084	0.260–11.570	0.035–0.204	0.728	213.40	/	/	/	/	/	/
**Sb**	0.213	8	91.8	/	0.033	/	0.264–0.807	/	0.352	/	0.288	0.29	0.92	**2.80**	7.2	8
**Se**	0.327 ^b^	0	100	ND	ND	ND	ND	ND	ND	/	/	/	/	/	/	390
**Y**	0.033 ^b^	97	0.0	5.458 ± 2.108	5.067	4.982	1.294–12.526	4.208–6.519	9.234	38.63	/	/	/	/	/	/

LoD = limit of detection (mg kg^−1^); *N* = number of samples above LoD; % cens = percentage of censored data; arithmetic mean results are presented as mean values ± S.D.; ND = not detected. First range numbers represent the minimum concentration detected. ^a^ Concentrations are presented as ng g^−1^. ^b^ LoD refers to the method detection limit in soil units, derived from the instrumental detection limit in the digestion solution (after digestion and final dilution) and back-calculated to soil concentration using sample mass and final volume. ^c^ Pollution indexes greater than the unit are marked in bold. ^d^ RGV established in soils in Aragón, Spain.

**Table 2:** Statistical summary of metal(loid)s in industrial soils of Alcalá (mg kg^−1^), their background values (BVs), coefficients of variation (CVs), pollution indices (PIs) and Regulatory Guidance Values (RGVs; mg kg^−1^) (BOCM, Order 761/2007).

Element	LoD	*N*	% Cens	Arithmetic Mean	Geometric Mean	Median	Range	Interquartile Range	P95	CV (%)	BV	Pollution Index			*PI* > 1%	RGV
												Mean	Min.	Max.		
**Ag**	0.049	9	59.1	0.066 ± 0.057	0.041	0.045	0.060–0.236	0.026–0.091	0.143	137.94	0.076	0.87	0.79	**3.13** ^c^	27.3	500
**Co**	0.059	22	0.0	3.811 ± 1.638	3.486	3.775	1.305–8.331	2.659–4.415	6.590	35.79	5.602	0.68	0.23	**1.49**	9.1	1500
**Fe**	0.189	22	0.0	10,287.7 ± 3780.95	9493.47	10,969.0	4278.3–15,821.9	7219.8–13,148.5	15,418.96	23.60	/	/	/	/	/	10,000 ^d^
**Mo**	0.282	1	95.5	/	0.005	/	0.854–0.854	/	<0.282	393.36	0.349	**2.45**	**2.45**	**2.45**	4.5	1500
**Pt ^a^**	0.345	15	31.8	0.944 ± 0.799	0.567	0.488	0.356–2.713	<0.345–1.369	2.474	53.50	/	/	/	/	/	/
**Rh ^a^**	0.246	5	77.3	0.168 ± 0.168	0.139	0.110	0.287–0.733	0.060–0.200	0.411	172.86	/	/	/	/	/	/
**Sb**	0.213	3	86.4	/	0.122	/	0.262–0.317	/	0.292	121.66	0.288	0.63	0.91	**1.10**	9.1	80
**Se**	0.327 ^b^	0	100	ND	ND	ND	ND	ND	ND	/	/	/	/	/	/	3900
**Y**	0.033 ^b^	22	0.0	8.119 ± 2.871	7.590	8.518	3.871–12.963	5.154–10.377	11.583	17.42	/	/	/	/	/	/

LoD = limit of detection (mg kg^−1^); *N* = number of samples above LoD; arithmetic mean results are presented as mean values ± S.D.; ND = not detected. First range numbers represent the minimum concentration detected. ^a^ Concentrations are presented as ng g^−1^. ^b^ LoD refers to the method detection limit in soil units, derived from the instrumental detection limit in the digestion solution (after digestion and final dilution) and back-calculated to soil concentration using sample mass and final volume. ^c^ Pollution indexes greater than the unit are marked in bold. ^d^ RGV established in soils in Aragón, Spain.

**Table 3:** Statistical summary of metal(loid)s in garden soils of Alcalá (mg kg^−1^), their background values (BVs), coefficients of variation (CVs), pollution indices (PIs) and Regulatory Guidance Values (RGVs; mg kg^−1^) (BOCM, Order 761/2007).

Element	LoD	*N*	% Cens	Arithmetic Mean	Geometric Mean	Median	Range	Interquartile Range	P95	CV (%)	BV	Pollution Index			*PI* > 1%	RGV
												Mean	Min.	Max.		
**Ag**	0.049	17	5.6	1.228 ± 1.694	0.507	0.439	0.085–5.397	0.214–1.726	5.356	115.85	0.076	**16.2** ^c^	**1.13**	**71.36**	94.4	5
**Co**	0.059	18	0.0	4.503 ± 1.611	4.277	4.076	2.510–9.343	3.536–4.999	7.029	26.06	5.602	0.80	0.45	**1.67**	16.7	15
**Fe**	0.189	18	0.0	10,202.33 ± 2408.11	9936.87	9788.129	5607.15–16,697.93	8987.37–11,317.62	13,206.41	18.69	/	/	/	/	/	3750 ^d^
**Mo**	0.282	5	72.2	0.362 ± 0.534	0.111	0.122	0.317–1.658	0.045–0.305	1.597	106.35	0.349	**1.04**	0.91	**4.75**	22.2	15
**Pt ^a^**	0.345	12	33.3	0.548 ± 0.293	0.425	0.409	0.368–1.312	<0.345–0.556	1.029	73.51	/	/	/	/	/	/
**Rh ^a^**	0.246	8	55.6	0.401 ± 0.617	0.210	0.168	0.343–2.557	0.074–0.419	1.484	60.23	/	/	/	/	/	/
**Sb**	0.213	5	72.2	0.191 ± 0.182	0.125	0.124	0.218–0.681	0.068–0.214	0.492	75.21	0.288	0.66	0.76	**2.36**	22.2	0.8
**Se**	0.327 ^b^	0	100	ND	ND	ND	ND	ND	ND	/	/	/	/	/	/	85
**Y**	0.033 ^b^	18	0.0	8.177 ± 1.424	8.066	7.745	6.184–11.750	7.369–9.103	10.236	17.42	/	/	/	/	/	/

LoD = limit of detection (mg kg^−1^); *N* = number of samples above LoD; arithmetic mean results are presented as mean values ± S.D.; ND = not detected. First range number represent the minimum concentration detected. ^a^ Concentrations are presented as ng g^−1^. ^b^ LoD refers to the method detection limit in soil units, derived from the instrumental detection limit in the digestion solution (after digestion and final dilution) and back-calculated to soil concentration using sample mass and final volume. ^c^ Pollution indexes greater than the unit are marked in bold. ^d^ RGV established in soils in Aragón, Spain.

**Table 4:** Non-carcinogenic risk derived from the oral exposure (HQ_oral_) to individual elements for each group of population considered.

Element	RfDo ^a^ (mg/kg × Day)	CDI_oral_ (mg/kg × Day)					HQ_oral_				
		Urban		Garden		Industrial	Urban Zone		Garden Zone		Industrial Zone
		Adults	Children	Adults	Children	Workers	Adults	Children	Adults	Children	Workers
**Ag**	5.00 × 10^−3^	1.33 × 10^−7^	2.48 × 10^−6^	8.77 × 10^−7^	1.64 × 10^−5^	8.85 × 10^−11^	2.66 × 10^−5^	4.96 × 10^−4^	1.75 × 10^−4^	3.27 × 10^−3^	1.77 × 10^−8^
**Co**	3.00 × 10^−4^	1.34 × 10^−6^	2.51 × 10^−5^	3.22 × 10^−6^	6.00 × 10^−5^	5.11 × 10^−9^	4.48 × 10^−3^	8.36 × 10^−2^	1.07 × 10^−2^	2.00 × 10^−1^	1.70 × 10^−5^
**Fe**	7.00 × 10^−1^	4.14 × 10^−3^	7.73 × 10^−2^	7.29 × 10^−3^	1.36 × 10^−1^	1.38 × 10^−5^	5.92 × 10^−3^	1.10 × 10^−1^	1.04 × 10^−2^	1.94 × 10^−1^	1.97 × 10^−5^
**Mo**	5.00 × 10^−3^	/	/	3.21 × 10^−7^	5.99 × 10^−6^	/	/	/	6.41 × 10^−5^	1.20 × 10^−3^	/
**Pt**	/	4.68 × 10^−7^	8.73 × 10^−6^	3.91 × 10^−7^	7.31 × 10^−6^	1.27 × 10^−9^	/	/	/	/	/
**Rh**	/	1.42 × 10^−7^	2.65 × 10^−6^	2.99 × 10^−7^	5.59 × 10^−6^	2.52 × 10^−10^	/	/	/	/	/
**Sb**	4.00 × 10^−4^	/	/	1.41 × 10^−7^	2.63 × 10^−6^	/	/	/	3.52 × 10^−4^	6.57 × 10^−3^	/
**Se**	5.00 × 10^−3^	/	/	/	/	/	/	/	/	/	/
**Y**	4.00 × 10^−3 b^	3.90 × 10^−6^	7.28 × 10^−5^	5.84 × 10^−6^	1.09 × 10^−4^	1.09 × 10^−8^	9.75 × 10^−4^	1.82 × 10^−2^	1.46 × 10^−3^	2.73 × 10^−2^	2.72 × 10^−6^

^a^ RfDo: oral reference dose [46] ^b^ RfDo for yttrium chloride [101].

**Table 5:** Non-carcinogenic risk derived from the inhalation exposure (HQ_inh_) to individual elements for each group of population considered.

Element	RfCi (mg/m^3^)	EC_inh_ (m^3^/µg)			HQ_inh_		
		Urban	Garden	Industrial	Urban	Garden	Industrial
		Adults		Workers	Adults		Workers
**Ag**	/	2.70 × 10^−6^	1.78 × 10^−5^	9.58 × 10^−7^	/	/	/
**Co**	6.00 × 10^−6^	2.73 × 10^−5^	6.54 × 10^−5^	5.53 × 10^−5^	4.55 × 10^−3^	1.09 × 10^−2^	9.22 × 10^−3^
**Fe**	/	8.42 × 10^−2^	1.48 × 10^−1^	1.49 × 10^−1^	/	/	/
**Mo**	2.00 × 10^−3^	/	6.52 × 10^−6^	/	/	3.26 × 10^−6^	/
**Pt**	/	9.51 × 10^−6^	7.96 × 10^−6^	1.37 × 10^−5^	/	/	/
**Rh**	/	2.89 × 10^−6^	6.08 × 10^−6^	2.73 × 10^−6^	/	/	/
**Sb**	3.00 × 10^−4^	/	2.86 × 10^−6^	/	/	9.53 × 10^−6^	/
**Se**	/	/	/	/	/	/	/
**Y**	/	7.93 × 10^−5^	1.19 × 10^−4^	1.18 × 10^−4^	/	/	/

## Data Availability

The original data presented in the study are openly available in Open Science Framework (OSF) at: https://osf.io/8wsjk/?view_only=8012ed923ba9422298a6a772a06070df (accessed on 15 January 2026).

## Supplementary Materials

The following supporting information can be downloaded at: https://www.mdpi.com/article/10.3390/jox16010017/s1, Table S1: Physicochemical characteristics of Alcalá de Henares’ soils for the three main areas monitored; Table S2: Concentration (mg kg^−1^) of a number of elements in Alcalá soil samples; Table S3: Calculated values of enrichment factor (*EF*) and percent of topsoil samples included in *EF* levels for each element per area monitored in Alcalá; Table S4: Factor loadings for varimax rotated PCA of metals data in Alcalá´s topsoils; Table S5: Factor loadings for varimax rotated PCA of metals data in Alcalá´s urban topsoils; Table S6: Evidence-based synthesis of dominant land-use patterns and plausible source interpretations for the monitored metal(loid)s in Alcalá de Henares topsoils; Figure S1: Spatial distribution of Ag, Co, Fe and Mo in Alcalá de Henares´s topsoils (n = 137). Concentrations are displayed using the class breaks shown in each panel legend (mg kg^−1^); Figure S2: Spatial distribution of Pt, Rh, Sb and Y in Alcalá de Henares´s topsoils (n = 137). Concentrations are displayed using the class breaks shown in each panel legend; Pt and Rh are expressed as ng g^−1^, whereas Sb and Y are expressed as mg kg^−1^; Figure S3: Pearson correlation coefficient matrix for metals in Alcalá de Henares´s topsoils (n = 137); Figure S4: Pearson correlation coefficient matrix for metals and other soils parameters in Alcalá de Henares´s topsoils (n = 137).
