# Supplementary material for: Geochemical Patterns and Human Health Risks of Less-Regulated Metal(loid)s in Historical Urban and Industrial Topsoils from Alcalá de Henares, Spain
Source: J Xenobiot. 2026 Jan 21;16(1):17. doi: 10.3390/jox16010017 (PMC12921956; doi:10.3390/jox16010017)
Supplement: Supplementary file 1 [file jox-16-00017-s001.zip › jox-4094131-supplementary.pdf]

Journal of Xenobiotics

Supplementary Materials: Geochemical patterns and human health risks of less-regulated metal(loid)s in historical urban and industrial topsoils from Alcalá de Henares, Spain.

Antonio Peña-Fernández, Manuel Higueras, Gevorg Tepanosyan, M. Ángeles Peña Fernández, M.C. Lobo

Table S1. Physicochemical characteristics of Alcalá de Henares' soils for the three main areas monitored.

Area	pH	E.C. (dS/m)	O.M. (%)	Sand (%)	Clay (%)	Silt (%)
**Urban**	7.81 ± 0.43[a]	830.08 ± 611.52[a]	1.96 ± 1.44[a]	45.33 ± 14.23[a]	17.40 ± 7.38[ab]	37.01 ± 11.73[a]
**Industrial**	8.09 ± 0.32[b]	276.77 ± 115.48[b]	1.43 ± 1.11[a]	24.5 ± 3.95[b]	17.50 ± 2.07[a]	58.0 ± 3.40[b]
**Garden**	7.68 ± 0.23[a]	830.10 ± 530.93[a]	4.90 ± 2.63[b]	37.17 ± 5.53[a]	22.17 ± 1.17[b]	40.83 ± 4.87[a]
**Pairwise Wilcoxon**	0.0131	0.00021	0.00147	0.000021	0.064	0.000032

Results (mean values ± S.D.) with different letter in the same column indicate significantly different values ($p < 0.05$). E.C.= electric conductivity; O.M.= organic matter content

Table S2. Concentration (mg kg^{-1}) of a number of elements in Alcalá soil samples.

Element	Urban zone	Industrial zone	Gardens	*P*
**Ag**	0.068 (<0.049-0.143) [a]	0.045 (0.026-0.091) [a]	0.439 (0.214-1.726) [b]	<0.0001
**Co**	1.577 (1.092-2.584) [a]	3.775 (2.659-4.415) [b]	4.076 (3.536-4.999) [b]	<0.0001
**Fe**	5,405.05 (3,319.15-7,818.30) [a]	10,969.0 (7,219.8-13,148.5) [b]	9,788.13 (8,987.37-11,317.62) [b]	<0.0001
**Mo**	0.295 *	0.554 **	0.122 (0.045-0.305)	/
**Pt** ✝	0.404 (<0.345-0.795)	0.488 (<0.345-1.369)	0.409 (<0.345-0.556)	0.195
**Rh** ✝	0.084 (0.035-0.204) [a]	0.110 (0.060-0.200) [ab]	0.168 (0.074-0.419) [b]	0.0408
**Sb**	0.352 *[a]	0.292 *[ab]	0.124 (0.068-0.214) [b]	0.0439
**Se**	ND	ND	ND	/
**Y**	4.982 (4.208-6.519) [a]	8.518 (5.154-10.377) [b]	7.745 (7.369-9.103) [b]	<0.0001

Results are presented as medians and IQR (all in mg kg^{-1}), ✝ except for Pt and Rh, which are presented in ng g^{-1}; ND= Not detected. Different letters in the same row indicate significantly different values (p-value < 0.05). * High sample percentil 95; ** high sample percentil 97.5.

Table S3. Calculated values of enrichment factor (EF) and percent of topsoil samples included in EF levels for each element per area monitored in Alcalá.

Area	Element	EF				Samples (%)					
		Mean	Min.	Max.	SD	EF < 2	2 < EF < 5	5 < EF < 20	20 < EF < 40	EF > 40	ED > 10[a]
Urb.	Ag	3.18	0.56	52.71	6.95	64.7	20.8	12.4	12.4	2.1	4.1
	Co	1.11	0.06	13.22	1.47	95.9	2.1	2.1	2.1	0.0	1.0
	Mo	0.42	0.91	10.36	2.15	96.2	2.7	1.0	1.0	0.03	0.4
	Sb	0.54	1.16	13.16	2.11	94.7	3.8	1.4	1.4	0.03	0.5
Ind.	Ag	0.64	0.52	2.30	0.52	97.0	3.0	0	0	0	0
	Co	1.20	0.98	1.83	0.25	100.0	0.0	0	0	0	0
	Mo	1.47	1.47	1.89	0.00	100.0	0.0	0	0	0	0
	Sb	0.37	0.71	3.11	0.73	97.7	2.3	0	0	0	0
Gar.	Ag	14.076	1.070	69.306	20.8	27.8	22.2	27.8	27.8	16.7	27.8
	Co	1.478	0.946	3.144	0.5	88.9	11.1	0	0	0	0
	Mo	1.066	0.983	6.109	1.8	86.7	8.9	4.4	4.4	0	0

	**Sb**	0.802	0.857	4.727	1.2		91.1	8.9	0	0	0	0

[a] EF > 10 indicates that that element has an anthropogenic origin.

Table S4. Factor loadings for varimax rotated PCA of metals data in Alcalá´s topsoils.

Element	PC1	PC2	PC3
**Ag**	**0.556**	0.299	-0.245
**Co**	**0.933**	-0.195	
**Fe**	**0.856**	-0.318	
**Mo**	0.269	**0.515**	0.356
**Pt**		-0.182	**0.914**
**Rh**	0.300	**0.679**	
**Sb**	0.268	**0.658**	
**Y**	**0.771**	-0.318	
**Eigenvalue**	2.748	1.523	1.041
**Variance, %**	34.346	19.034	13.013
**Cumulative, %**	34.346	53.380	66.393

In bold: strong (> 0.7) and moderate (0.5–0.7) loadings.

Table S5. Factor loadings for varimax rotated PCA of metals data in Alcalá´s urban topsoils.

Element	PC1	PC2	PC3
**Ag**	**0.605**		-0.438
**Co**	**0.905**		
**Fe**	**0.821**	-0.273	
**Mo**		0.469	**0.720**
**Pt**	-0.422		0.228
**Rh**	0.260	**0.766**	
**Sb**	0.266	**0.634**	-0.438
**Y**	**0.658**		
**Eigenvalue**	2.646	1.401	1.038
**Variance, %**	33.074	17.508	12.972
**Cumulative, %**	33.074	50.582	63.554

In bold: strong (> 0.7) and moderate (0.5–0.7) loadings.

Table S6. Evidence-based synthesis of dominant land-use patterns and plausible source interpretations for the monitored metal(loid)s in Alcalá de Henares topsoils.

Element	Main observed pattern in this dataset	Primary line(s) of evidence used here	Most plausible source/control in Alcalá context (hypothesis-based)
Ag	Strong enrichment in gardens; hotspots; high PI/EF	Land-use contrast + EF/PI + hotspot behaviour	Managed-soil inputs (compost/biosolids/soil imports) and/or localised urban inputs; requires confirmatory tracers
Co	Higher in industrial; low censoring; relatively coherent distribution	Descriptive statistics + PCA (geogenic association)	Predominantly lithogenic, with possible industrial contribution in industrial estates
Fe	Elevated in industrial (and some gardens)	Descriptive statistics + land-use contrast	Mostly lithogenic, with industrial/urban dust contributions in impacted areas
Mo	Highly censored but locally elevated (some gardens/industrial)	Censored-data summaries + PCA association with anthropogenic component	Diffuse urban/traffic-related or local point inputs; definitive attribution requires source sampling
Sb	More evident in urban and some garden samples; censored overall	Land-use contrast + PCA (anthropogenic component)	Traffic-related non-exhaust (brake wear) and urban materials

Pt	Low levels; loads mainly on PC3	PCA (PC3 dominance) + spatial patterns	Traffic/catalyst-related PGE emissions (hypothesis supported by PGE literature)
Rh	Low levels; censored; aligns with anthropogenic component	PCA (PC2), censored statistics + spatial patterns	Traffic/catalyst and/or localised inputs; uncertainty due to censoring
Y	Detected in all; EF near unity (crust-normalised); higher industrial/garden than urban	EF approach + PCA (geogenic association)	Mostly geogenic; limited anthropogenic deposition possible

This table consolidates, for each monitored element, (i) the main concentration pattern observed across land uses (urban parks/urban zone, industrial zone and gardens), (ii) the primary lines of evidence used in this study to support interpretation (land-use contrasts, enrichment/pollution metrics, multivariate structure and spatial hotspot mapping), and (iii) cautious, hypothesis-based interpretations of the most plausible sources/controls in the local context. Interpretations are evidence-proportionate and do not constitute definitive source apportionment, which would require targeted source sampling and/or additional tracers. Abbreviations: EF = enrichment factor (dimensionless indicator of enrichment relative to a reference element/background); PI = pollution index (index used to describe relative contamination status; see Methods for definition); PCA = principal component analysis; PC1, PC2, PC3 = principal components 1–3 (latent factors extracted by PCA); PGE(s) = platinum-group element(s) (here including Pt and Rh); LoD = limit of detection (analytical).

Figure S1. Spatial distribution of Ag, Co, Fe and Mo in Alcalá de Henares´s topsoils (n=137). Concentrations are displayed using the class breaks shown in each panel legend (mg kg⁻¹).

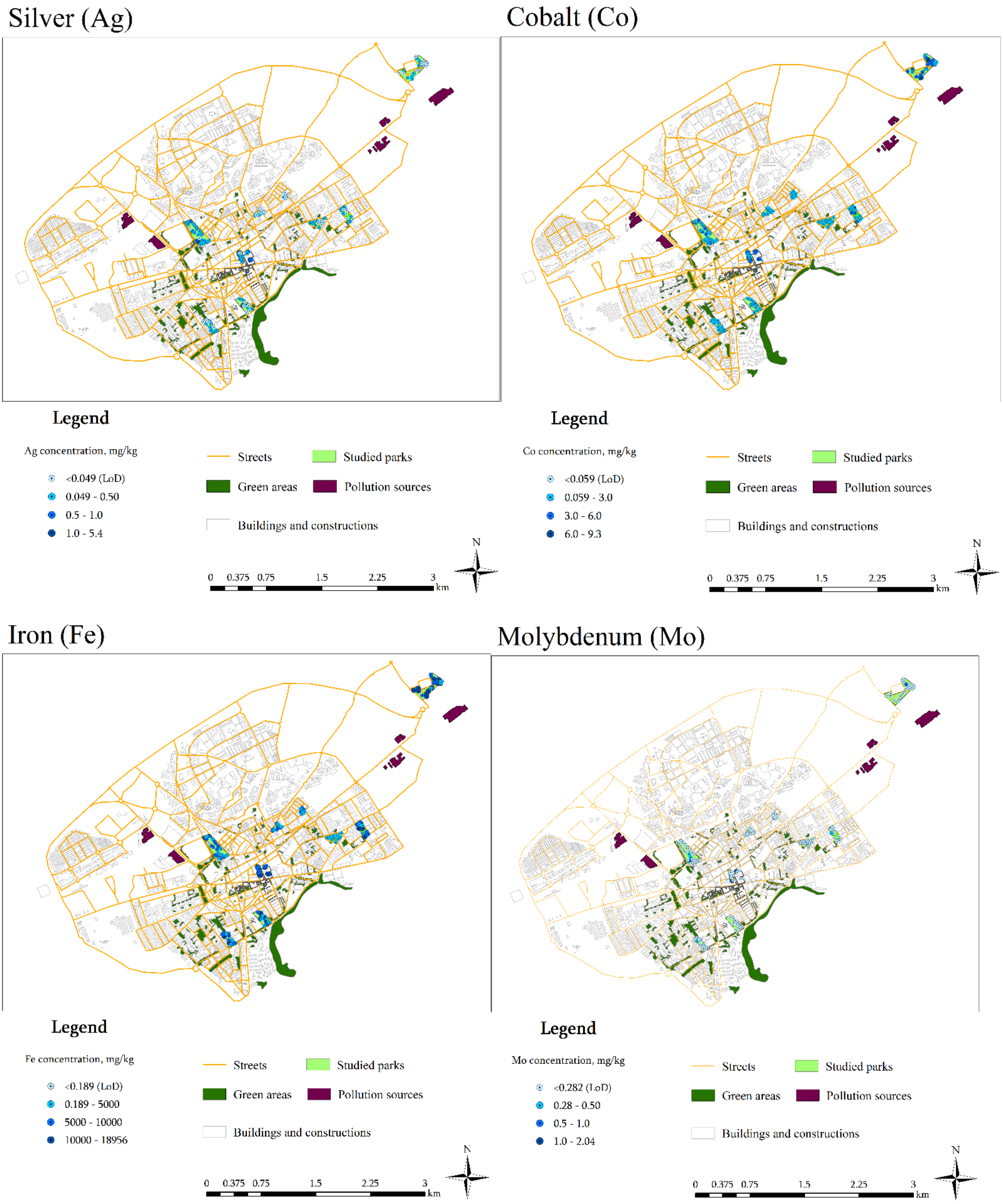

Figure S2. Spatial distribution of Pt, Rh, Sb and Y in Alcalá de Henares´s topsoils (n=137). Concentrations are displayed using the class breaks shown in each panel legend; Pt and Rh are expressed as ng g^{-1}, whereas Sb and Y are expressed as mg kg^{-1}.

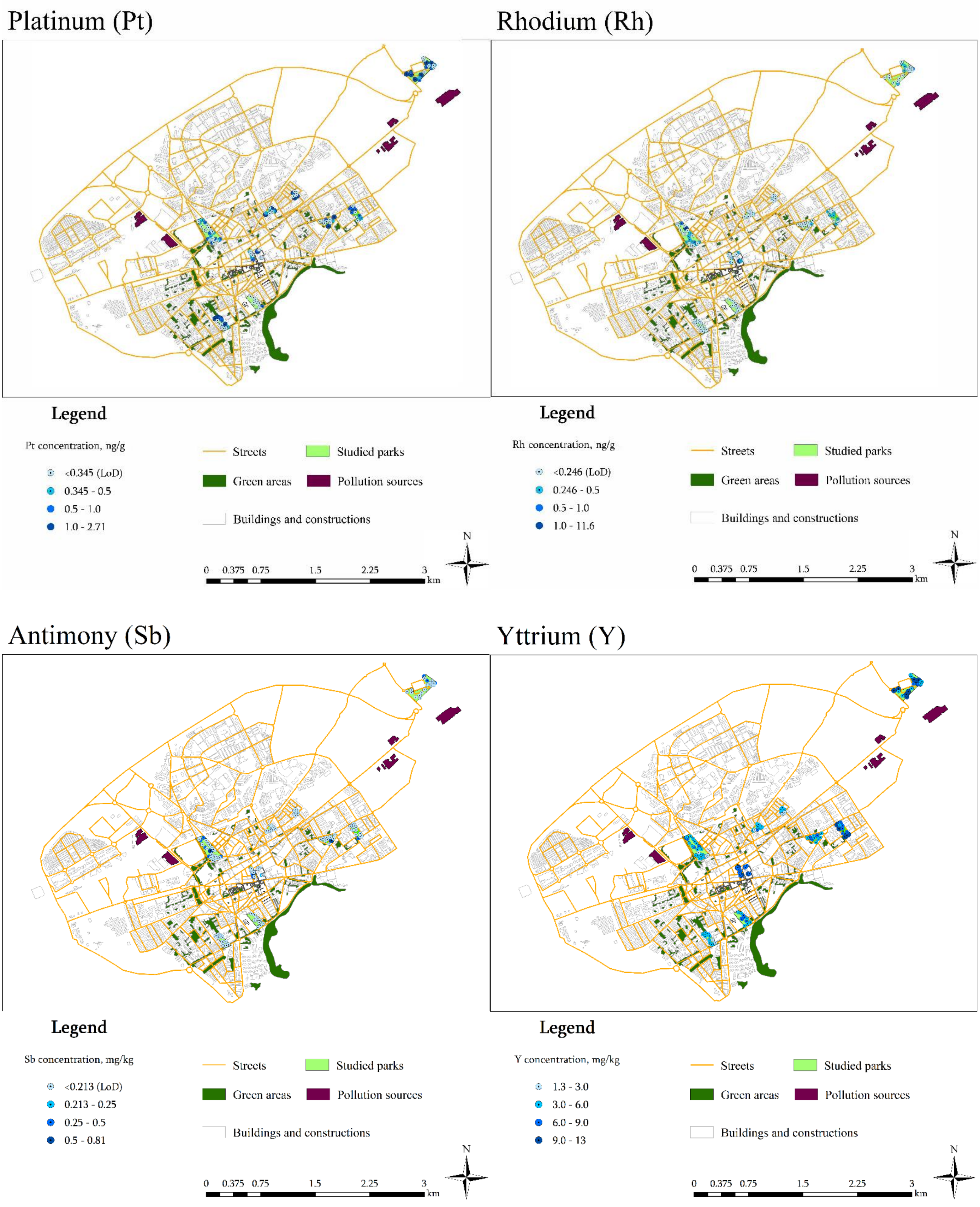

Figure S3. Pearson correlation coefficient matrix for metals in Alcalá de Henares´s topsoils (n=137).

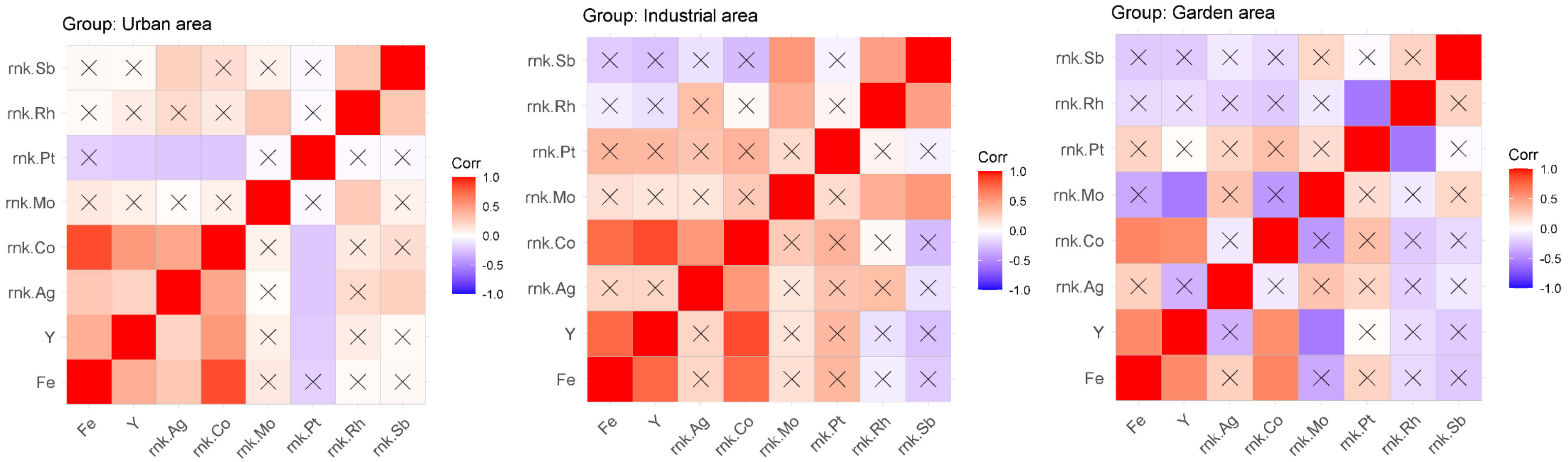

Coloured cells represent Pearson correlation coefficients (r) between pairs of variables. Warmer colours indicate stronger positive correlations, and cooler colours indicate negative correlations. Crosses indicate correlation estimates that are not statistically significant ($p \geq 0.05$, two-tailed Pearson test); cells without crosses correspond to significant correlations at $p < 0.05$.

Figure S4. Pearson correlation coefficient matrix for metals and other soils parameters in Alcalá de Henares´s topsoils (n=137).

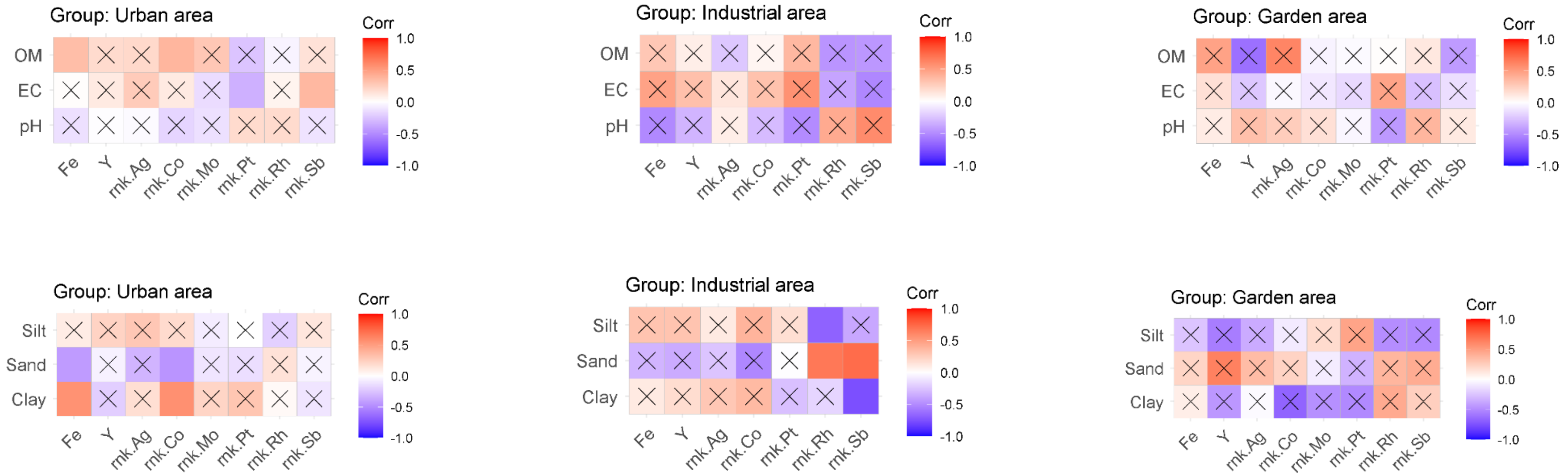

Coloured cells represent Pearson correlation coefficients (r) between pairs of variables. Warmer colours indicate stronger positive correlations, and cooler colours indicate negative correlations. Crosses indicate correlation estimates that are not statistically significant ($p \geq 0.05$, two-tailed Pearson test); cells without crosses correspond to significant correlations at $p < 0.05$.
